# The Current Status of the Disease Caused by Enterovirus 71 Infections: Epidemiology, Pathogenesis, Molecular Epidemiology, and Vaccine Development

**DOI:** 10.3390/ijerph13090890

**Published:** 2016-09-09

**Authors:** Ping-Chin Chang, Shou-Chien Chen, Kow-Tong Chen

**Affiliations:** 1 Division of Infectious Disease, Department of Internal Medicine, Chi-Mei Medical Center, Liouying, Tainan 736, Taiwan; 2LH101@TMH.org.tw; 2 Department of Family Medicine, Da-Chien General Hospital, Miaoli 237, Taiwan; scchen818@gmail.com; 3 General Education Center, Ta Tung University, Taipei 104, Taiwan; 4 Department of Occupational Medicine, Tainan Municipal Hospital, Tainan 701, Taiwan; 5 Department of Public Health, College of Medicine, National Cheng Kung University, Tainan 701, Taiwan

**Keywords:** hand-foot-mouth disease, herpangina, enterovirus 71, pathogenesis, virology

## Abstract

Enterovirus 71 (EV71) infections have a major public health impact in the Asia-Pacific region. We reviewed the epidemiology, pathogenesis, and molecular epidemiology of EV71 infection as well as EV71 vaccine development. Previous studies were found using the search terms “enterovirus 71” and “epidemiology” or “pathogenesis” or “molecular epidemiology” or “vaccine” in Medline and PubMed. Articles that were not published in the English language, manuscripts without an abstract, and opinion articles were excluded from the review. The reported epidemiology of cases caused by EV71 infection varied from country to country; seasonal variations in incidence were observed. Most cases of EV71 infection that resulted in hospitalization for complications occurred in children less than five years old. The brainstem was the most likely major target of EV71 infection. The emergence of the EV71 epidemic in the Asia-Pacific region has been associated with the circulation of different genetic lineages (genotypes B3, B4, C1, C2, and C4) that appear to be undergoing rapid evolutionary changes. The relationship between the gene structure of the EV71 virus and the factors that ensure its survival, circulation, and evasion of immunity is still unknown. EV71 infection has emerged as an important global public health problem. Vaccine development, including the development of inactivated whole-virus live attenuated, subviral particles, and DNA vaccines, has been progressing.

## 1. Introduction

Hand, foot, and mouth disease (HFMD) is a common childhood disease caused by enteroviruses. A proportion of HFMD patients develop severe neurological complications and die [1]. Enteroviruses are members of the family of single positive-stranded ribonucleic acid (RNA) viruses called *Picornaviridae* [1]. Initially, enteroviruses were classified into five types, including polioviruses, coxsackie group A viruses (types 1–22, 24), coxsackie group B viruses (types 1–6), echoviruses (types 1–7, 9, 11–27, 29–34), and enteroviruses (types 68–71) [1]. Human enteroviruses are divided into four species, including human enterovirus A (HEV-A), HEV-B, HEV-C, and HEV-D, based on homology within the RNA region coding for the VP1 capsid protein. Recently, many new enterovirus serotypes have been characterized by molecular methods, bringing the number of known serotypes to more than 100 within 12 species [1,2]. 

Enterovirus 71 (EV71) is one of the major etiologic agents of HFMD and herpangina [3]. EV71 was first isolated from a child with encephalitis in California in 1969 [4]. Since that time, several epidemics have been reported [5,6]. The spectrum of EV71 infection is wide and includes cutaneous, visceral, and neurological manifestations. In recent years, EV71 was known to cause several large-scale outbreaks of severe complications in children involving the central nervous system (CNS). Although the virus is present in most countries, outbreaks of the disease have been predominantly found in the Asia-Pacific region; the reasons for this phenomenon are unclear [3,7,8,9,10,11,12,13,14,15,16]. Because of the potential of the virus to cause severe neurologic disease, we need to understand the characteristics of EV71 infection. The aim of this study was to explore the epidemiology, pathogenesis, molecular epidemiology, and prospective of vaccine development of EV71.

All papers published from January 1965 through August 2016 describing patients affected by enterovirus 71 were obtained by searching Medline (National Library of Medicine, Bethesda, MD, USA) using the keyword “enterovirus 71” and “epidemiology” or “pathogenesis” or “molecular epidemiology” or “vaccine”. Articles not published in the English language were excluded from the review. Updated information on enterovirus 71 infection in Taiwan was obtained from the Taiwan Centers for Disease Control (Taiwan CDC) [17].

The articles were reviewed, and only reports of original studies were retained; manuscripts without an abstract (assumed not to be original), review articles, opinion articles, etc., were excluded. After selecting the articles, relevant information was extracted and classified with respect to the basic science (epidemiology, virology), the clinical indicators (symptomatology, visits to the emergency department, and hospitalization), the information source (laboratory, and surveillance), the year of publication, and the study design. 

Searches were performed in July and August 2016. A total of 1235 documents were retrieved from Medline. After screening the articles, 195 were considered to be relevant. Eighty-five percent of the studies were retrospective studies, 10% were perspective studies, and 5% used other study designs.

## 2. Epidemiology

The EV71 epidemic was reported in the 1970s by various countries in America, Europe, Australia, and Asia [4,18,19,20,21]. 

After the EV71 epidemics occurred in Australia and Japan in the 1970s [19,20], further small epidemics and sporadic clusters were reported in Hong Kong in 1985 [22] and in Australia in 1986 (Table 1) [3,9,10,14,15,19,20,21,22,23,24,25,26,27,28,29,30,31,32,33,34,35,36,37,38,39,40,41,42,43,44,45,46,47,48,49]. In 1997, a large epidemic of EV71 occurred in Sarawak, Malaysia [9]. Around the same time, an EV71 outbreak with four fatal cases was reported in peninsular Malaysia [24], and several cases of severe neurological disease were reported in Japan [14]. In 1998, the largest EV71 epidemic occurred in Taiwan [10]. There were at least 405 children hospitalized with serious neurological complications, of whom 78 died. Recently, the latest large Asian-Pacific epidemic was in China in 2008, when approximately 490,000 infections and 126 deaths of children were reported [15]. In addition to these epidemics, a pattern of a 2- to 3-year cyclic epidemic has been found in many areas, including Japan [25], Sarawak [26], and Taiwan [3].

Outside the Asia-Pacific region, EV71 circulates at a low level in the regions of Canada, the USA, Europe, and Africa and causes sporadic cases or small outbreaks (Table 2) [6,50,51,52,53,54,55,56,57,58,59,60,61,62]. In 1975, there was a large epidemic of an EV71 infection in Bulgaria, in which 140 cases resulted in paralysis and 27 cases were fatal [50]. In 1978, an epidemic of EV71 occurred in Hungary, in which the major clinical manifestation included meningitis and encephalitis [6]. During 1998, 20 children with EV71 were hospitalized in Canada [51], in which half of the cases had aseptic meningitis, and one-third of the cases had respiratory symptoms. All of the patients recovered rapidly. In the USA, there were two small community outbreaks of EV71 infection with neurological involvement in 2003 and 2005 [52]. These outbreaks affected 16 children aged younger than nine years old, and only one child died. Between 2001 and 2004, 9% of the children hospitalized with aseptic meningitis were found to be infected with EV71 in Austria [53]. During 1998 and 2006, thirty-two cases of EV71 infection with neurological disease, HFMD, or both were identified in the UK [54]. In 2007, after 21 years of low endemicity, 58 patients were hospitalized with EV71 infection and CNS involvement in the Netherlands [55]. Widespread circulation of EV71 was also reported between October 2002 and October 2003 in Norway [56], where EV71 was isolated from 19 (17%) of 113 healthy children. In addition, the detection rate for any enterovirus was low (less than 1 per 4000 people) in a screening study over 22 months in Scotland [57]. In Nairobi, Kenya, two small outbreaks of EV71 infection in HIV treatment centers were reported in 1999 and 2000 [58].

**Figure 1:**
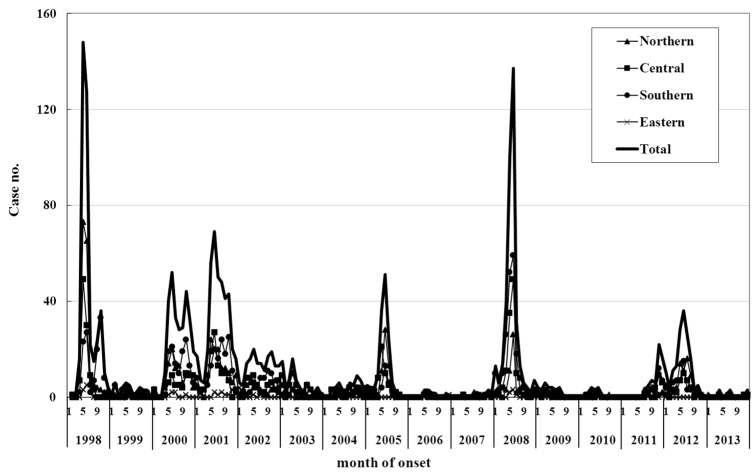
The number of cases of severe hand, foot, and mouth disease and herpangina in Taiwan, overall and by region, 1998–2013.

A higher incidence was observed during the summer months in Asia [63,64,65], and epidemics recur with a seasonal pattern. Some studies have also reported a variation of the peak season between different years [25,66]. In Taiwan, a surveillance system was established at the Taiwan Centers for Disease Control (Taiwan CDC) in 1998 to assess the epidemiologic features of EV71 infection [17]. Patients who were hospitalized for HFMD/herpangina were reported to the Taiwan CDC [3,17]. From March 1998 through December 2013, epidemic peaks occurred every year, with the highest number of cases occurring during the summer season (Figure 1). Most cases of HFMD occurred in children aged five years old or younger; males had a higher incidence rate of HFMD than females. 

Current hypotheses explaining the seasonal pattern of EV71 infection include host immune competence fluctuations mediated by seasonal factors, such as melatonin or vitamin D levels [67]; seasonal, behavioral factors unrelated to weather, such as school attendance and indoor crowding [68]; environmental factors [63,65], including temperature and relative humidity. However, human behavioral factors alone do not appear to account for the seasonal pattern observed for certain cases of EV71 infection, including cases that occur in school-aged children or in association with household crowding [68]. The relationship between meteorological data and the incidence of EV71 infection has been investigated [69,70]. A study by Chang et al. [70] showed that the incidence of EV71 infection reflected significant summer seasonality from April to June in Taiwan. The incidence of EV71 infection began to increase at a temperature above 13 °C; at a temperature higher than approximately 26 °C, the incidence began to decline, producing an inverted V-shaped relationship. This study indicated that warmer temperatures and elevated humidity would lead to an increased rate of EV71 infection in Taiwan. Similar findings have been found in China [71,72]. It showed that the HFMD infections occurred with two seasonal peaks, in summer (June) and winter (November or December). The major spatial-temporal clusters were from the eastern coastal and southern regions. The risk of infection was relatively high at 10 °C ≤ *t* < 15 °C and 15 °C ≤ *t* < 20 °C, and peaked at 20 °C ≤ *t* < 25 °C. The distribution of pathogens’ serotypes and the level of sunshine could be risk factors for the outbreak of HFMD in China. 

HFMD is spread from person to person by contact with saliva, respiratory secretions, fluid in vesicles, and feces. Transmission of HFMD can be reduced by isolation of confirmed cases of patients with viral shedding, and maintaining good hygiene, including hand-washing and disinfection of surfaces in child care settings [73].

Taken together, EV71 infection has emerged as an important public health problem in the world, especially in the Asia-Pacific region. The morbidity and mortality of EV71 infection are influenced by geographic, host, and environmental factors. Understanding the epidemiology and transmission dynamics of EV71 is helpful for leading to the control and elimination EV71 infection. 

## 3. Pathogenesis

EV71 infection that causes brainstem encephalitis, especially affecting the medulla and associated with cardiopulmonary dysfunction, has become a notable feature in EV71 epidemics in Asia and is associated with high mortality. The most common manifestation of enteroviral infection is a non-specific febrile illness lasting three days, followed by recovery. However, some cases progress to severe or fatal illness. Brainstem encephalitis, especially affecting the medulla and associated with cardiopulmonary dysfunction, has been noted as a clinical feature in EV71 epidemics in Asia and is an important cause of death [5,12,15,69,74,75]. 

Viremia occurred more frequently in children under the age of one year [76,77]. However, viremia did not have a clear significant effect on the clinical severity of EV71 infection. Additionally, the frequency of patients with CNS involvement was similar between patients with or without viremia [77,78]. 

EV71 is a highly neurotropic virus, and the brain stem is the most common target of EV71 infection [10,24,74,79,80,81]. Similar to poliovirus, two likely routes by which the EV71 virus involves the CNS have been considered: the virus is either transmitted to the CNS from the blood across the blood-brain barrier (BBB) or enters the CNS through peripheral nerves via retrograde axonal transport [82,83,84,85].

The strong neurotropism of EV71 and retrograde axonal transport in neurons might represent the major transmission route of EV71 in mice [86,87]. In another study, mice were infected via the oral and parenteral routes with a murine-adapted virus strain that originated from a fatal human case, and the results showed that the EV71 virus entered the CNS via peripheral motor nerves after a skeletal muscle infection and spread within the CNS through motor and other neural pathways [88]. In a study of an autopsy sample in Malaysia, inflammation was found to be the most marked in the spinal cord gray matter, brainstem, hypothalamus, and subthalamic and dentate nuclei [89]. 

Neurological virulence is one of the most severe complications responsible for death [52]. The mechanism of the neurological complications of EV71 infection is poorly understood. Cordey et al. [90] have reported that an L97R alteration in the VP1 protein enhances the neuronal tropism of EV71. Some alterations in VP1, 5’ NCR, and protease 2A affect viral virulence [91]. These results suggested that sequence variations may contribute to neural infection and neurological complications.

What is the mechanism of pulmonary edema among severe cases with EV71 infection? It may be due to the destruction of the medial, ventral, and caudal medullas, which may lead to sympathetic overactivation, causing a blood shift to the lungs [92,93]. Similar to acute respiratory distress syndrome, the pulmonary edema that occurs in children with EV71 brainstem encephalitis may be caused by abnormal cytokine activation that produces a severe inflammatory response, which in turn causes increased pulmonary vascular permeability [94]. One study showed that children with severe EV71 encephalitis were significantly more likely to have a cytotoxic T lymphocyte antigen haplotype (CTLA-4) than children who did not contract severe EV71 infection [95].

In summary, some EV71 strains are often related with neurological manifestations but EV71 also causes aseptic meningitis and fever without a source with good outcomes in very young infants. Some of the epidemiologic studies include cases of children with non-severe manifestations [96,97]. Moreover, there is a bias when analyzing some of the clinical data due to the fact that severe outbreaks are often more easily reported [98]. Based on the data from autopsy studies of fatal cases with EV71 infection in Taiwan [74,99,100,101], peninsular Malaysia [24], and Hong Kong [100], there is evidence that brainstem encephalitis due to EV71 infection is sufficient to cause neurogenic pulmonary edema. During the viral life cycle, enteroviruses are influenced by viral factors and multiple host factors. The combination of the interactive effect of the virus and the host is vital for viral replication, virulence, and pathogenicity [102]. However, tissue-specific viral virulence remains unclear in both cell-based systems and animal models and requires further investigation in the future [102]. 

## 4. Molecular Epidemiology

The phylogenetic origins of the EV71 strains recently circulating in the Asia-Pacific region have been studied (Table 1) [3,9,10,14,15,19,20,21,22,23,24,25,26,27,28,29,30,31,32,33,34,35,36,37,38,39,40,41,42,43,44,45,46,47,48,49]. Using the VP1 protein for analysis, EV71 can be divided into four distinct genogroups (A, B, C, and D) [29,38]. Genogroups B and C can be further divided into genogroups B1–B5 and C1–C5, respectively [39]. Recently, genogroup D was identified in India, and genogroups E and F were identified in Africa [38,40]. Genogroup A comprises the prototype EV71 strain (BrCr-CA-70), which was isolated in 1969 in the United States [4] but had not been detected subsequently until 2008 [15]; however, the source of the virus in this outbreak is unclear [103]. In contrast, genogroup B and C viruses have been causing large-scale epidemics in Asia since 1997 and are targeted for vaccine development [41,42]. The co-circulation of four distinct genogroups (B3, B4, C1, and C2) in Malaysia from 1997–2000 has been well documented [36]. Singapore [43] and Western Australia [104] were also affected by the B3 genogroup from 1997–1999. In Taiwan, different serotypes of EV71 are in circulation; predominant genogroups occurred in 1998 (C2), 2000–2001 (B4), 2004–2005 (C4), 2008–2009 (B5), 2010–2011 (C4), and 2011–2012 (B5) [41,44,45]. 

In South Korea, an EV71 outbreak was reported during 2009 [28]. The predominant genotype was C4, particularly C4a, which was associated with relatively low severity and a low case-fatality rate [28]. However, in China, it has been reported that the mean evolution rate of C4a EV71 is faster than all other EV71 genotypes [48]. The evolutionary branch C4a has some crucial nucleotide or amino acid mutations relative to branch C4b, and these changes may be responsible for its increased neurovirulence and the epidemic of large-scale outbreaks of HFMD in China [48]. EV71 genotype C4a viruses also spread from China to Vietnam and caused a large-scale epidemic in Ho Chi Minh City and southern Vietnam in 2011, which was confirmed through genetic and antigenic analysis [4]. Based on phylogenetic analyses of the VP1 sequences, a comprehensive evolutionary dynamic study of EV71 from 1994–2013 was conducted in the Asia-Pacific areas [49]. They showed that C4, C1, C2, and B4 are the predominant strains, and the polymorphisms and divergence of the VP1 gene of the EV71 strains evolve very slowly, which may be one of the reasons for periodic outbreaks in this area. 

The phylogenetic origins of the EV71 strains circulating outside the Asia-Pacific region have been studied (Table 2) [6,50,51,52,53,54,55,56,57,58,59,60,61,62]. An epidemiology study of EV71 infections in France from 2000–2009 showed that C1 isolates were predominant between 1994 and 2005, and C2 strains have been predominant since 2007 [59]. Additional epidemiological and genetic data on EV71 circulation in the Netherlands from 1963 to 2008 were analyzed [55]. Infections were caused by three different and successive lineages belonging to subgenotypes B0, B1, and B2 from 1963 to 1986. After 1987, the B genotype was replaced by genotype C strains of lineages C1 and C2. The epidemiology of the EV71 subgenotypes B1, B2, C1, and C2 appeared to have a global nature [55]. A large-scale genetic analysis of isolates was collected from 19 countries worldwide over a 40-year period [60]. A series of recombination events occurred over the study period and was identified through incongruities in the sequence grouping between the VP1 and 3D pol regions. The likelihood of recombination increased with VP1 sequence divergence, and recombination events occurred as an initiation of most subgenotypes immediately preceding their lineage expansion and global emergence [6,50,51,52,53,54,55,56,57,58,59,60,61,62].

EV71 has a high mutability and is in constant evolution, similar to poliovirus [29]. However, to what extent genetic exchanges explain the variations in biological or epidemic behavior is open to debate. Genogroups B and C have been associated with both complicated and uncomplicated disease [35,99], thus making it difficult to pinpoint a virus-specific marker of virulence [53,105]. EV71 lacks the use of a DNA template for correcting mismatches, resulting in an average of one mutation per new genome copy [106]. Furthermore, genomic recombination is frequently used among enteroviruses as a mechanism to produce variants [107], presumably as a response to selection pressure [108]. These observations suggest that recombination and mutation may promote the spread of EV71 in the human population [109].

Taken together, the dynamics of the genetic and antigenic evolution of EV71 in the past decade showed a genotype shift with an antigenic property change and genome recombination. Almost all HFMD outbreaks were correlated with genetic variations caused by EV71 switches. Recombination of EV71 in the region encoding the nonstructural proteins is frequently observed, and numerous recombination crossover breakpoints have been identified within the non-structural genes, particularly of the more recent EV71 subgenotypes [44,110,111]. Recombination has also been observed between EV71 and coxsackievirus A16 [110,112] and other enterovirus A species [44]. Interestingly, recent studies have identified several subgenotype-specific recombination events [60,113] that appear to act as founding events in subgenotype emergence and global expansion [60], suggesting that recombination has played a crucial role in EV71 evolution. Thus, continual monitoring of antigenic variation and genetic evolution is critical for epidemic control and vaccine design.

## 5. Vaccine Development

EV71 is most commonly transmitted via close person-to-person contact; however, the majority of EV71 infections are asymptomatic or result in mild disease, which limits the effectiveness of public health interventions such as hand washing. The development of an effective vaccine may be the best way to control EV71 infection [114]. Many EV71 vaccines have been investigated, including an inactivated virus vaccine [115,116,117], a virus-like particle vaccine [118], DNA vaccines [119], a subunit vaccine [120], and a live attenuated vaccine [121]. 

Inactivated whole-virus EV71 vaccines are the most advanced candidates among the current EV71 vaccines. Inspired by previous experiences in developing inactivated vaccines, the development of inactivated whole-virus EV71 vaccines is progressing rapidly [122]. The formaldehyde-inactivated EV71 vaccines effectively protected animals from lethal virus challenge in several animal model studies [123,124]. Additionally, a formalin-inactivated EV71 virion formulated in alum-adjuvant vaccine resulted in satisfactory cross-neutralizing antibody responses in a phase III trial [115]. Phase III clinical trials of inactivated EV71 vaccines have been completed in China, involving more than 30,000 infants and children [104,125,126,127]. Their results have shown that the safety of the EV71 vaccine is satisfactory in infants and children and can prevent over 90% of EV71-associated HFMD and 80% of EV71-associated disease [125,127]. In December 2015, China’s Food and Drug Administration approved two inactivated EV-A71 vaccines for preventing severe HFMD [128]. Similar vaccines (e.g., formalin-inactivated EV71 vaccines) are being developed in Taiwan and Singapore [126,128], both of them have entered Phase I clinical trials. Inactivated EV71 vaccines, due to their inability to replicate, are preferred over the live attenuated vaccines for safety reasons. However, the cost of production of inactivated vaccines and potential supply problems cause substantial frustration in practical implementations [129]. 

The development of virus-like particles (VLPs) vaccines is a different approach from the inactivated whole-virus EV71 vaccines. VLPs vaccines provide the advantage of presenting all surface epitopes of the EV71 capsid proteins in their native conformations at once [128,130]. Although VLPs vaccines have a lower efficacy than inactivated vaccines, experimental animal model studies have indicated that VLPs can generate protective neutralizing antibodies and are cross-reactive against multiple subtypes that are not in the vaccine [130]. The main problem associated with VLPs is their stability, purification, and cost of manufacturing. The other types of EV71 vaccines (DNA vaccines, subunit vaccine, and live attenuated vaccine) are in the early stages of development, with the most advanced undergoing preclinical trials in mice and non-human primates [128].

In summary, the formalin-inactivated EV71 vaccines have reported to have high efficacy for preventing EV71 infection. EV71-related HFMD could become a vaccine-preventable disease in the world. However, no formalin-inactivated EV71 vaccines protect against CAV16, which is predominantly responsible for annual HFMD outbreaks. The development of vaccines that cover multiple species of enterovirus is a prospective option. Live attenuated vaccines, subunits vaccine, synthetic peptides, and DNA vaccines have been approached by researchers, but these approaches are in the early stage of vaccine development.

## 6. Conclusions

EV71 has emerged as a significant infection in the Asia-Pacific region. It is a potentially fatal neurotropic virus that may be considered the new polio. Several factors have been indicated to regulate EV71 replication. However, specific factors that contribute to neural pathogenesis remain unclear. Further study on the mechanism of EV71 infection is still needed. Although inactivated EV71 vaccines have been rapidly developed in the last few years, these vaccines have some limitations. Other types of vaccines are being developed to prevent EV71 infection. A global EV71 infection surveillance network should be established, and continuous epidemiological surveillance is important for identifying and detecting the potential emergence of new EV71 variants. 

## Figures and Tables

**Table 1:** A summary of the human enterovirus 71 genotypes circulating in the Asia-Pacific region by country, 1997–2012 [3,9,10,14,15,19,20,21,22,23,24,25,26,27,28,29,30,31,32,33,34,35,36,37,38,39,40,41,42,43,44,45,46,47,48,49].

Countries	1997	1998	1999	2000	2001	2002	2003	2004	2005	2006	2007	2008	2009	2010	2011	2012
Singapore	B3, B4	B3, C1	B3	B4	B4	C1, B4	-	-	B5	B5	B5	B5	-	-	-	-
Malaysia	B3, **B4**	C1, **B4**	-	**B4**, C1	**B4**, C1	-	**C1**, B4, B5	B5	B5	B5	**-**	-	**-**	**-**	**-**	**-**
Australia	B3	B3	B3, C2	C1	-	-	C1	C1	-	-	-	-	-	-	-	-
Japan	B3, B4, C2	C2	C2	B4	B4	C2	B5	-	C4a	C4a	**C2**, C4a	**C2**	**C2**	**C2**	-	-
Korea	-	-	-	C3	-	-	C4b	-	-	-	-	-	**C2**, C4a	-	-	-
Taiwan	-	**C2**, B4	B4	B4	B4	B4	B4	C4	C5	B5	B5	B5	B5	C4	C4	B5
China	-	-	-	-	-	-	-	-	-	-	-	C4	C4	C4	C4	-

Bold type indicates predominant; -, no data available.

**Table 2:** A summary of human enterovirus 71 genotypes circulating outside the Asia-Pacific region, 1960–2012 [6,50,51,52,53,54,55,56,57,58,59,60,61,62].

Countries	1960–1969	1970–1979	1980–1989	1990–1999	2000–2009	2010	2011	2012
France	-	-	-	-	C1, C2, C4	-	-	C4
UK	-	-	-	C1	C1, C2	-	-	-
Germany	-	-	-	-	C1, C2	-	-	-
Austria	-	-	-	-	C1, C4	-	-	-
Norway	-	-	-	-	C1	-	-	-
Netherlands	B0	B1	B2	C1	C1, C2	-	-	-
Hungary	-	B1	-	-	C1, C4	-	-	-
Bulgaria	-	B1	-	-	-	-	-	-
USA	A	B1	B2	C1, C2	C2	-	-	-
Canada	-	-	-	-	-	-	-	-

-, no data available.
